# POCUS to Evaluate for Achilles Tendon Involvement in Ankle Lacerations

**DOI:** 10.24908/pocusj.v11i01.20121

**Published:** 2026-04-22

**Authors:** Ana Ruiz Castaneda, Gretel Rodriguez, Olivia Peña, Paul Khalil

**Affiliations:** 1 Department of Emergency Medicine, Nicklaus Children's Hospital, Miami, FL, USA; 2 Department of Pediatric Urgent Care, Nicklaus Children's Hospital, Miami, FL, USA; 3 Duke University, Durham, NC, USA

**Keywords:** Achilles tendon rupture, POCUS, Ankle lacerations, Point of care ultrasound

## Abstract

Achilles tendon injury is uncommon in children but is often caused by direct trauma to the posterior ankle. The diagnosis of Achilles tendon involvement after injury may be inadequate when based solely on physical examination findings. There is limited literature describing the identification of Achilles tendon injury in the pediatric population with point of care ultrasound (POCUS). This case series describes two patients with ankle lacerations in whom Achilles tendon involvement was evaluated using POCUS.

## Case Presentation

### Case 1

**Figure 1.**
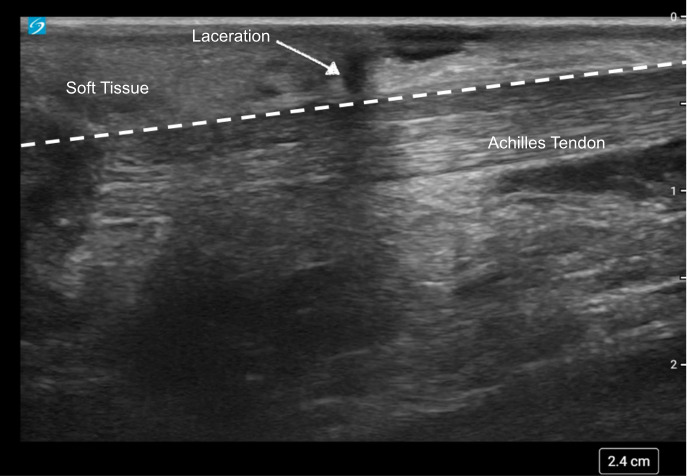
Point of care ultrasound (POCUS) image showing a sagittal view of the Achilles tendon (below the dotted line) with a laceration (arrow) above, but not including, the tendon.

A 9-year-old girl presented in the emergency department with a right foot laceration over the medial calcaneus that occurred three hours prior to arrival. She was performing cartwheels at home when, upon landing, she grazed the medial aspect of her ankle against a door latch. She was unable to bear weight on the right foot secondary to pain. Vital signs included blood pressure 132/86 mmHg, temperature of 98.8°F, heart rate of 92 beats/min, respiratory rate of 24 breaths/min, and oxygen saturation of 99%. A physical exam revealed that she had a 5 cm jagged laceration injury extended laterally from the medial process of the calcaneus towards the lateral process. She had good range of motion of the leg, foot, and phalanges. There was no numbness, tingling, or weakness of the lower extremity. Point of care ultrasound (POCUS) was performed, demonstrating an intact Achilles tendon (Figure 1). The patient followed up as an outpatient for suture removal with good range of motion and without complications.

### Case 2

**Figure 2.**
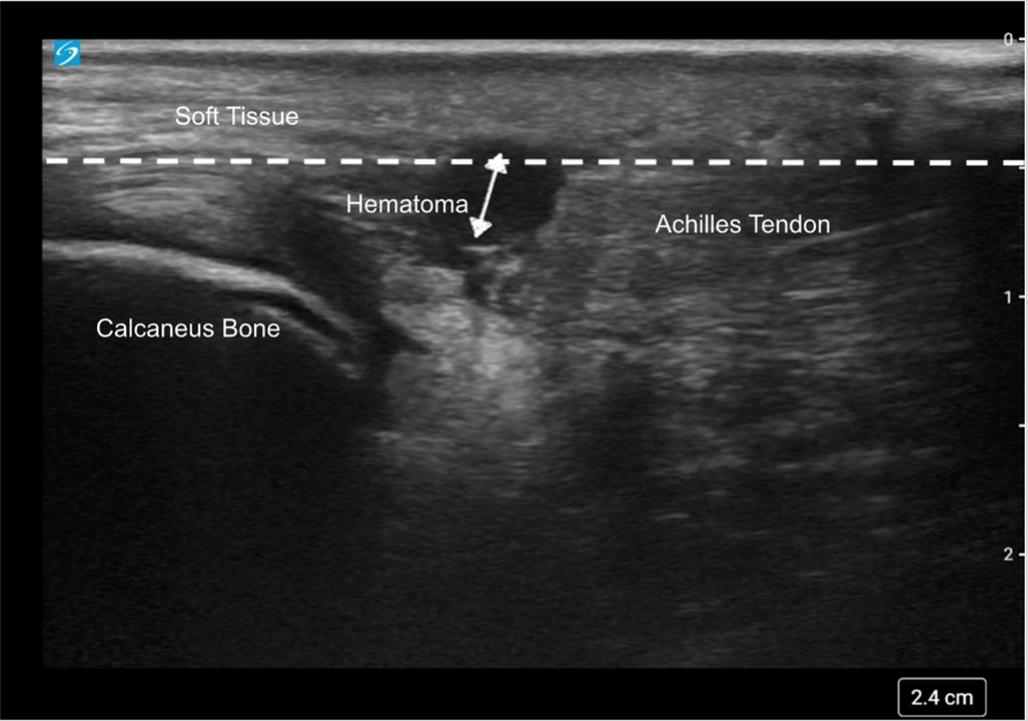
Point of care ultrasound (POCUS) image showing a sagittal view of a lacerated Achilles tendon (tendon below the dotted line) with a hematoma (double arrows above the laceration).

A 10-year-old girl presented for right ankle pain due to sustained injury just prior to arrival, where a metal gate was accidentally closed on her right foot. She was able to walk on her toes but with difficulty secondary to pain. Vital signs included blood pressure 120/85 mmHg, heart rate of 82 beats/min, respiratory rate of 24 breaths/min, and oxygen saturation of 99%. A physical exam showed a 5 × 3 cm triangular-shaped laceration on the posterior aspect of the right ankle, horizontally overlying the Achilles tendon, with active bleeding. She was able to perform dorsiflexion and plantarflexion of the right foot, however, she endorsed pain with movement. The right dorsalis pedis pulse was intact. POCUS showed laceration of the Achilles tendon (Figure 2). Orthopedic surgery was consulted and recommended skin closure, immobilization with crutches, and discharge with follow-up. She later returned to the emergency department for persistent pain and was taken to the operating room for wound washout and repair.

## POCUS Findings

In both cases, a high-frequency linear probe (L12-3) on a Sonosite PX ultrasound machine was used to examine the ankle. Case 1 showed a laceration superior to the Achilles tendon with no evidence of tendon involvement on sagittal imaging (Figure 1). Case 2 showed a laceration to the Achilles tendon with a hematoma on sagittal imaging (Figure 2).

## Technique

**Figure 3.**
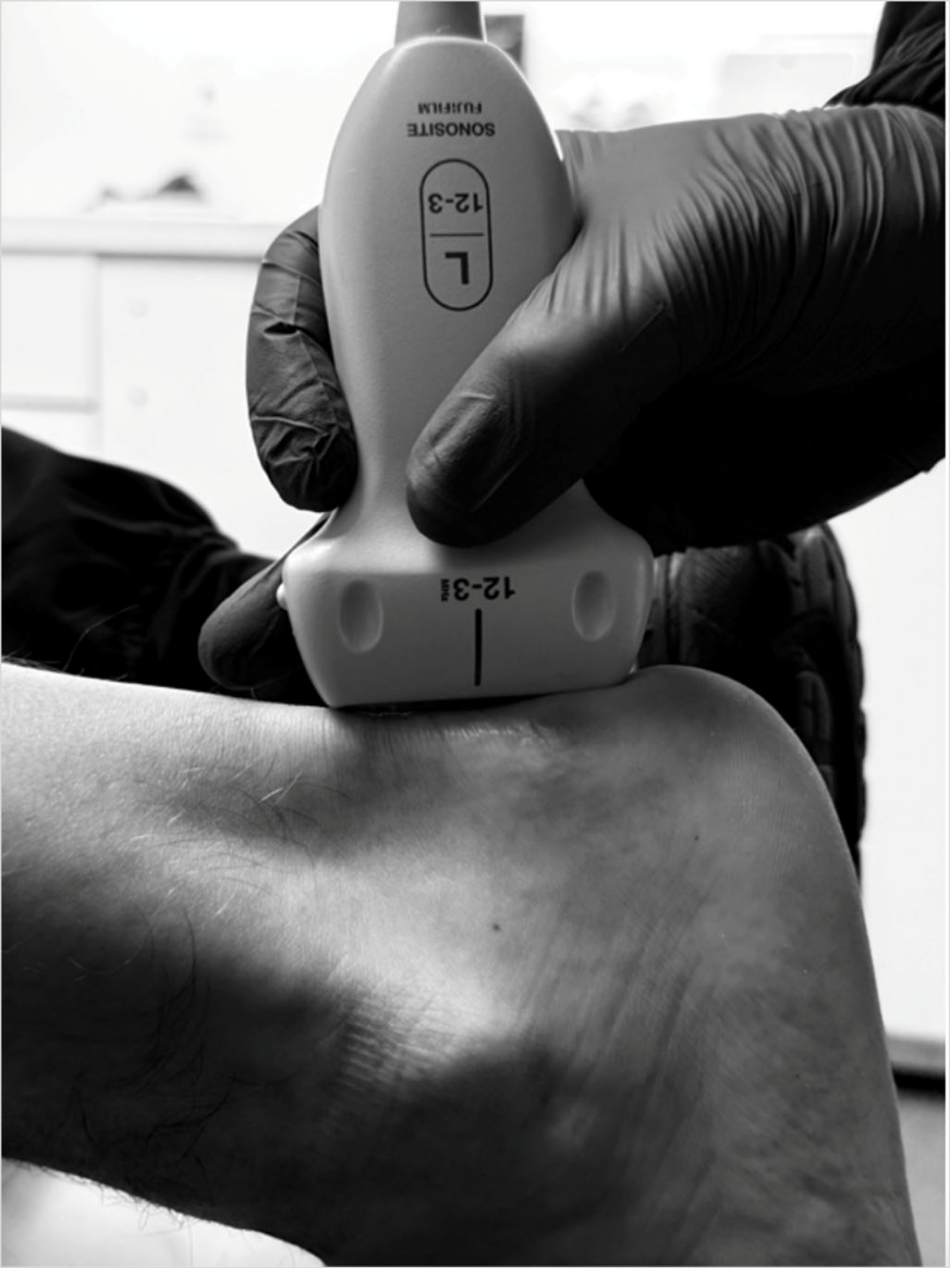
The sagittal view is obtained by placing the ultrasound probe maker toward the patient's head and scanning from the calcaneal tuberosity to the distal calf.

A high-frequency linear transducer should be used to image the laceration and the Achilles tendon. Prior to scanning, the probe should be properly cleaned and covered with either a Tegaderm or a sterile transducer cover, as a laceration is an open wound. Sterile gel should be used. The patient should be scanned while lying in prone position with their feet hanging off the bed, if possible. The wound should be scanned in its entirety, along with the Achilles tendon. The Achilles tendon should be scanned in the transverse and sagittal planes using both static and dynamic imaging techniques. The sagittal view can be obtained by placing the probe maker toward the patient's head and scanning from the calcaneal tuberosity to the distal calf. The transverse view can be obtained by turning the probe so that it is perpendicular to the Achilles tendon and scanning from the calcaneal tuberosity to the distal calf (Figure 3). During dynamic scanning, the patient should be instructed to dorsiflex the foot. Lacerations to the tendon will have disruption of the fibrillary appearance.

## Discussion

Achilles tendon injury is uncommon in children [1]. In one study, from 2012–2016 in the United States, the largest overall incidence of Achilles tendon ruptures occurred in patients ages 20–39 years [1]. Evaluation was conducted by physical examination and showed that the Thompson test and observation of a gap in the normal contour of the tendon are useful but limited tools. Overlying lacerations of the posterior heel also have the potential to mask tendon injuries [2]. One case report by Vasileff & Moutzouros evaluated a 10-year-old boy in whom a posterior heel laceration masked the diagnosis of Achilles tendon injury and led to a subsequent complete Achilles tendon rupture that required open tendon repair [1]. Early diagnosis of Achilles tendon injury is critical. When Achilles tendon injury goes unrecognized, it can lead to prolonged pain and limitations of activity [3].

Medical resonance imaging (MRI) was long thought to be the imaging modality of choice for evaluating an Achilles tendon injury. Over the past decade, multiple studies have shown that ultrasonography is a reasonable alternative tool. In one systematic review of the literature that included 56 studies, ultrasound was recommended over MRI for diagnosing and monitoring an Achilles tendon injury [4]. The study further noted the utility of POCUS for identifying other clinical information about the injury [4].

Several studies and case reports have examined the use of ultrasonography for the evaluation of an Achilles tendon injury. Adhikari et al. described a case in which Achilles tendon injury was successfully diagnosed by POCUS [5]. High-frequency color Doppler ultrasonography (HFCDU) was studied by Lui et al. in 68 patients with suspected Achilles tendon injury [6]. HFCDU evaluation of the laceration showed the Achilles tendon was swollen and thickened, with reduced echogenicity and blurred fiber texture [6]. Analysis of the data showed a sensitivity of 98% in diagnosis of Achilles tendon injury [6]. Another pediatric POCUS case series described POCUS for Achilles tendon rupture [7]. To our knowledge, no other studies examined use of POCUS after open Achilles tendon injuries or for Achilles tendon lacerations in children.

## Conclusion

In our pediatric cases, POCUS was used to evaluate Achilles tendon involvement when direct trauma occurred to the posterior heel secondary to lacerations. Identification of Achilles tendon involvement allowed for the appropriate and timely consultation of orthopedic specialists from the time of injury. There is limited research about the evaluation of Achilles tendon with POCUS, particularly after direct trauma and laceration of the posterior heel. More studies—specifically in the pediatric population—are needed to further evaluate this.
